# Aberrant JAK-STAT signaling-mediated chromatin remodeling impairs the sensitivity of NK/T-cell lymphoma to chidamide

**DOI:** 10.1186/s13148-023-01436-6

**Published:** 2023-02-06

**Authors:** Jinghong Chen, Zhixiang Zuo, Yan Gao, Xiaosai Yao, Peiyong Guan, Yali Wang, Zhimei Li, Zhilong Liu, Jing Han Hong, Peng Deng, Jason Yongsheng Chan, Daryl Ming Zhe Cheah, Jingquan Lim, Kelila Xin Ye Chai, Burton Kuan Hui Chia, Jane Wan Lu Pang, Joanna Koh, Dachuan Huang, Haixia He, Yichen Sun, Lizhen Liu, Shini Liu, Yuhua Huang, Xiaoxiao Wang, Hua You, Sahil Ajit Saraf, Nicholas Francis Grigoropoulos, Xiaoqiu Li, Jinxin Bei, Tiebang Kang, Soon Thye Lim, Bin Tean Teh, Huiqiang Huang, Choon Kiat Ong, Jing Tan

**Affiliations:** 1 State Key Laboratory of Oncology in South China, Collaborative Innovation Center of Cancer Medicine, Sun Yat-Sen University Cancer Center, 651 East Dongfeng Road, Guangzhou, 510060 China; 2 Institute of Molecular and Cell Biology, Singapore, Singapore; 3 Cancer and Stem Cell Biology Program, Duke-NUS Medical School, Singapore, Singapore; 4 Laboratory of Cancer Epigenome, Division of Medical Sciences, National Cancer Centre Singapore, Singapore, Singapore; 5 Department of Hematology, Southwest Hospital, Third Military Medical University, Chongqing, China; 6 Division of Medical Oncology, National Cancer Centre Singapore, Singapore, Singapore; 7 Cancer Science Institute of Singapore, National University of Singapore, Singapore, Singapore; 8 Lymphoma Genomic Translational Research Laboratory, Cellular and Molecular Research, National Cancer Centre Singapore, 11 Hospital Drive, Singapore, 169610 Singapore; 9 Affiliated Cancer Hospital & Institute of Guangzhou Medical University, Guangzhou, China; 10 Department of Anatomical Pathology, Singapore General Hospital, Singapore, Singapore; 11 Department of Hematology, Singapore General Hospital, Singapore, Singapore; 12 Department of Pathology, Fudan University Shanghai Cancer Center, Shanghai, China

**Keywords:** NKTL, HDAC inhibitor, Chidamide resistance, JAK-STAT pathway, Chromatin remodeling

## Abstract

**Background:**

Natural killer/T-cell lymphoma (NKTL) is a rare type of aggressive and heterogeneous non-Hodgkin's lymphoma (NHL) with a poor prognosis and limited therapeutic options. Therefore, there is an urgent need to exploit potential novel therapeutic targets for the treatment of NKTL. Histone deacetylase (HDAC) inhibitor chidamide was recently approved for treating relapsed/refractory peripheral T-cell lymphoma (PTCL) patients. However, its therapeutic efficacy in NKTL remains unclear.

**Methods:**

We performed a phase II clinical trial to evaluate the efficacy of chidamide in 28 relapsed/refractory NKTL patients. Integrative transcriptomic, chromatin profiling analysis and functional studies were performed to identify potential predictive biomarkers and unravel the mechanisms of resistance to chidamide. Immunohistochemistry (IHC) was used to validate the predictive biomarkers in tumors from the clinical trial.

**Results:**

We demonstrated that chidamide is effective in treating relapsed/refractory NKTL patients, achieving an overall response and complete response rate of 39 and 18%, respectively. In vitro studies showed that hyperactivity of JAK-STAT signaling in NKTL cell lines was associated with the resistance to chidamide. Mechanistically, our results revealed that aberrant JAK-STAT signaling remodels the chromatin and confers resistance to chidamide. Subsequently, inhibition of JAK-STAT activity could overcome resistance to chidamide by reprogramming the chromatin from a resistant to sensitive state, leading to synergistic anti-tumor effect in vitro and in vivo. More importantly, our clinical data demonstrated that combinatorial therapy with chidamide and JAK inhibitor ruxolitinib is effective against chidamide-resistant NKTL. In addition, we identified TNFRSF8 (CD30), a downstream target of the JAK-STAT pathway, as a potential biomarker that could predict NKTL sensitivity to chidamide.

**Conclusions:**

Our study suggests that chidamide, in combination with JAK-STAT inhibitors, can be a novel targeted therapy in the standard of care for NKTL.

*Trial registration*: ClinicalTrials.gov, NCT02878278. Registered 25 August 2016, https://clinicaltrials.gov/ct2/show/NCT02878278

**Supplementary Information:**

The online version contains supplementary material available at 10.1186/s13148-023-01436-6.

## Introduction

Natural killer/T-cell lymphoma (NKTL) is an aggressive Epstein–Barr virus-associated T-cell lymphoma (TCL) that accounts for 10% of all TCLs [1–3]. It is more prevalent in East Asia and Latin America and is histologically characterized by angiodestruction, prominent necrosis and a cytotoxic phenotype [4–6]. Current therapeutic strategies for newly diagnosed advanced and relapsed/refractory NKTL include asparaginase-based systematic chemotherapies with or without hematopoietic stem cell transplantation. However, the long-term survival rate for this population of patients remains low, at 40–52% [7, 8]. There is thus an urgent need to exploit potential novel therapeutic targets for the treatment of NKTL.

Histone acetylation is an important mode of epigenetic regulation controlled by the antagonistic actions of two classes of enzymes: histone acetyltransferases (HATs) and histone deacetylases (HDACs). HATs acetylate histones, leading to open chromatin regions that promote gene transcription. On the other hand, HDACs deacetylate histones, resulting in chromatin condensation, which induces transcriptional repression [9]. Frequent somatic mutations in genes related to epigenetic regulation have been identified in NKTL, such as mutations of *EP300*, *MLL2*, *ARID1A* and *BCOR* [10, 11]. Aberrant HAT/HDAC expression or abnormal activity can promote cancer cell proliferation and survival, which may play an important role in NKTL pathogenesis [12]. Inhibiting HDACs could therefore be a potential novel strategy for NKTL treatment.

Multiple HDAC inhibitors, such as vorinostat (SAHA), romidepsin (FK-228) and belinostat, have shown promising clinical efficacy in TCL patients [13]. Approximately 30% of cutaneous T-cell lymphoma (CTCL) patients were found to respond to SAHA and FK-228 [14, 15]. Chidamide is a novel and orally active benzamide-class HDAC inhibitor that selectively blocks the activities of HDAC1, HDAC2, HDAC3 and HDAC10, inducing growth arrest and apoptosis in certain cancers [16, 17]. It belongs to a unique class of HDAC inhibitors distinct from vorinostat and romidepsin, having a different chemical structure. In a multicenter clinical trial, chidamide monotherapy achieved a remarkable objective response rate (ORR) of 39% in 256 relapsed/refractory peripheral T-cell lymphoma (PTCL) patients, confirming the clinical efficacy of HDAC inhibitors in this disease [18]. Chidamide was approved by the National Medical Products Administration (NMPA) to treat patients with relapsed/refractory PTCL in 2014 in China. More recently (2021), approval has also been granted for the treatment of relapsed/refractory adult T-cell leukemia (ATL) in Japan by the Pharmaceuticals and Medical Devices Agency (PMDA). Currently, chidamide as a monotherapeutic agent for diffuse large B-cell lymphoma (DLBCL) is being evaluated in a phase II clinical trial in Japan and in combination with nivolumab as a first-line therapy for melanoma in a phase III clinical trial in the USA.

In this study, we evaluated the therapeutic effects of chidamide in 28 relapsed/refractory NKTL patients, and a remarkable ORR of 39% was achieved. We identified potential biomarkers to predict the clinical response to chidamide. Moreover, we deciphered the underlying mechanisms of primary resistance to chidamide mediated by aberrant JAK-STAT signaling and found that JAK-STAT inhibitors can effectively overcome this resistance in the treatment of NKTL.

## Methods

### Patients and cell lines

A phase II clinical trial to evaluate chidamide monotherapy in relapsed/refractory NKTL was conducted at Sun Yat-sen University Cancer Center (SYSUCC). The study was approved by the institutional review board of SYSUCC in accordance with the Declaration of Helsinki, and applicable regulations according to Good Clinical Practice guidelines. Written informed consent was obtained from all patients before enrollment in the trial. This trial was registered at ClinicalTrials.gov, number NCT02878278. Research protocols for tumor collection and analysis were approved by the ethical committees of SYSUCC. The details for the patients and cell lines are described in the Supplementary Methods. We routinely tested mycoplasma contamination in cell culture using Myco-Blue® Mycoplasma Detector (Vazyme) monthly.

### Drugs

Chidamide was provided by Shenzhen Chipscreen Biosciences Ltd. SAHA was obtained from Sigma–Aldrich. Trichostatin A (TSA), belinostat and tofacitinib were purchased from Selleckchem. Romidepsin was obtained from MedChemExpress. Ruxolitinib and Stattic were purchased from Axon Medchem and Millipore, respectively.

### Cell viability assays

A total of 2000 cells were seeded in 96-well plates in triplicate and treated with the indicated drugs at various concentrations or not treated for 96 h. Cell viability was measured by the CellTiter-Glo Luminescent Cell Viability Assay (Promega) according to the manufacturer’s instructions, and luminescence was read using an Infinite M200 plate reader (Tecan, Switzerland). Half maximal inhibitory concentration (IC50) values were calculated, and proliferation curves were plotted using GraphPad Prism.

### Calculation of the combination index (CI)

CI values were determined by the inhibition rate of the cells treated with drugs and computed using CalcuSyn [19]. CI values greater than, equal to, and less than 1 indicate antagonism, additivity and synergism, respectively.

### Cell cycle assays

2 × 10^5^ cells were treated with indicated drugs for 72 h. Cells were washed with cold PBS and fixed with 70% ethanol for more than 1 h. Fixed cells were washed with cold PBS twice and treated with 100 μl 100 μg/ml ribonuclease A (RNase A, TAKARA) for 5 min. The cells were then stained with 400 μl 50 μg/ml propidium iodide (PI, Thermo Fisher Scientific) for 40 min in the dark and analyzed by flow cytometry (SP6800, Sony).

### Cell apoptosis assay

2 × 10^5^ cells were treated with indicated drugs for 72 h. Cells were washed with ice-cold PBS and stained with Annexin V-FITC Apoptosis Detection Kit (Vazyme) following the manufacturer’s instructions. The stained cells were detected using flow cytometry (SP6800, Sony).

### H&E staining and immunohistochemistry (IHC)

H&E staining, IHC for TNFRSF8 (CD30) and Epstein–Barr encoding region in situ hybridization (EBER ISH) were performed with formalin-fixed, paraffin-embedded (FFPE) tissues from NKTL patients using standard procedures [20, 21] with a TNFRSF8 kit (Cell Signaling Technology) and the Bond Ready-to-Use ISH EBER Probe kit (Leica Biosystems), respectively. CD30 expression in at least 20% of tumor cells was considered positive, and samples were scored by at least two expert pathologists.

### ChIP-qPCR, ChIP-seq and ChIP-seq data analyses

The primer sequences used for ChIP-qPCR are listed in Additional File 2: Table S1. ChIP-seq was performed as previously described, with slight modifications [22]. The details are described in the Supplementary Methods.

### Real-time RT–qPCR, RNA-seq and RNA-seq data analyses

The primer sequences used for RT–qPCR are listed in Additional File 2: Table S2. The methods for real-time RT–qPCR, RNA-seq and RNA-seq data analyses are described in the Supplementary Methods.

### In vivo studies

In vivo studies were conducted in compliance with animal protocols approved by the SingHealth Institutional Animal Care and Use Committee. Five- to eight-week-old female NOD/SCID/IL2rγnull (NSG) mice were kept under standard laboratory conditions according to the National Advisory Committee for Laboratory Animal Research guidelines. The details are provided in the Supplementary Methods.

### Statistical analysis

Graphs include results that are expressed as the mean ± SD of three independent experiments. Statistical differences between the two groups were evaluated by a two-tailed Student’s *t test*, while significant differences among multiple groups were analyzed by two-way ANOVA with Bonferroni’s correction using GraphPad Prism 7. Fisher’s exact test was used to assess the association between protein expression and clinical response. *p* < 0.05 was considered statistically significant.

## Results

### Clinical efficacy of chidamide, a novel HDAC inhibitor, in relapsed/refractory NKTL patients

**Fig. 1:**
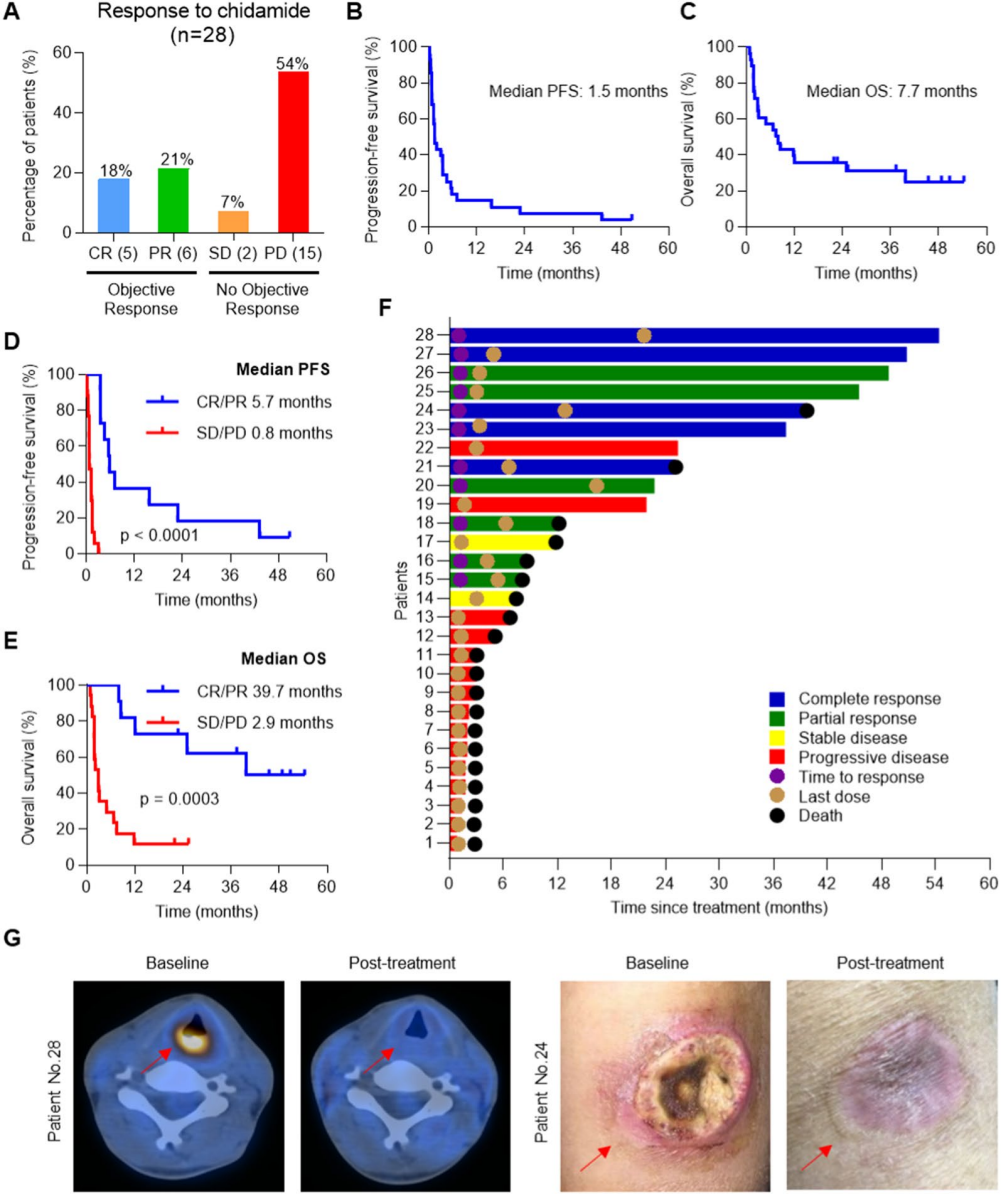
Efficacy of chidamide in 28 relapsed/refractory NKTL patients. **A** Assessment of responses in 28 relapsed/refractory NKTL patients treated with chidamide. CR (5), complete response, 5 patients; PR (6), partial response, 6 patients; SD (2), stable disease, 2 patients; PD (15), progressive disease, 15 patients. Patients with CR or PR were classified as having objective responses, while those with SD or PD were classified as not having objective responses. **B** Kaplan–Meier curve for PFS of all patients treated with chidamide. **C** Kaplan–Meier curve for OS of all patients treated with chidamide. **D** Kaplan–Meier curve comparing the PFS of patients who responded to chidamide (*n* = 11) with the PFS of those who did not respond (*n* = 17); p value, log-rank test. **E** Kaplan–Meier curve comparing the OS of patients who responded to chidamide (*n* = 11) with the OS of those who did not respond (*n* = 17); p value, log-rank test. **F** Swimmer plot showing duration since treatment initiation and clinical outcomes for patients. **G** Representative images showing tumor lesions (red arrow) before and after chidamide treatment in two relapsed/refractory NKTL patients who achieved CR. Upper images, positron emission tomography-computed tomography (PET-CT) imaging of patient No. 28, throat; lower images, photographs of patient No. 24, left arm

To evaluate the efficacy of chidamide, a single-arm, phase II clinical trial was performed in 28 NKTL patients who relapsed after or were refractory to chemoradiotherapy and/or autologous stem cell transplantation (ASCT). The patients were treated with chidamide until disease progression or intolerance. The demographic and clinical characteristics of the 28 patients are summarized in Additional File 2: Tables S3 and S4. Among the 28 patients, 5 patients (18%) achieved complete response (CR) and 6 patients (21%) achieved partial response (PR), with an objective response rate (ORR) of 39%. Two patients (7%) had stable disease (SD), and 15 patients (54%) had progressive disease (PD) (Fig. 1A). The median progression-free survival (PFS) and median overall survival (OS) of the 28 NKTL patients were 1.5 months and 7.7 months, respectively (Fig. 1B, C). Both the median PFS and OS of patients with CR or PR were significantly superior to those of patients with SD or PD (median PFS, 5.7 months vs. 0.8 months, *p* < 0.0001, log-rank test; median OS, 39.7 months vs. 2.9 months, *p* = 0.0003, log-rank test) (Fig. 1D, E). The time to response and clinical outcomes were summarized in a swimmer plot (Fig. 1F). Analysis of representative images of tumor lesions before and after chidamide treatment demonstrated that chidamide produced a favorable clinical response in relapsed/refractory NKTL patients (Fig. 1G). Despite the good clinical outcome in 39% of the NKTL patients treated, the remainder of the patients did not benefit from chidamide treatment, indicating primary resistance. Therefore, there is an urgent need to uncover predictive biomarkers to enable patient stratification and explore potential therapeutic strategies to alleviate resistance to chidamide therapy.

### Characterization of NKTL cell line sensitivity to chidamide in vitro

**Fig. 2:**
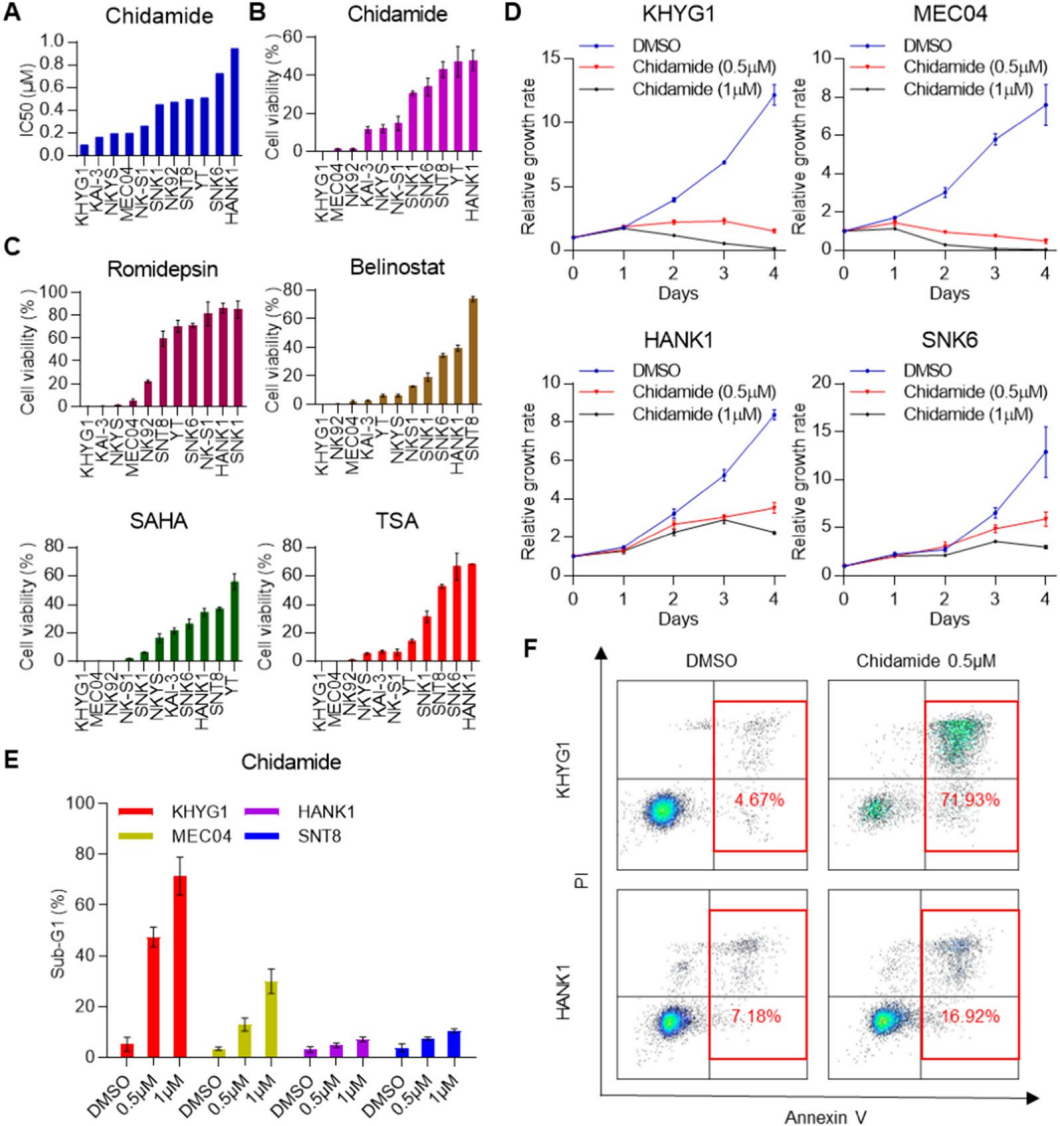
Chidamide inhibits cell proliferation and selectively induces the death of NKTL cells. **A** The effect of chidamide on the proliferation of 11 NKTL cell lines at 96 h posttreatment, represented as the concentration of chidamide required to inhibit proliferation by 50% (IC50). **B–C** Cell viability responses of NKTL cells to the HDAC inhibitors chidamide (1 μM), romidepsin (2.5 nM), belinostat (400 nM), SAHA (1 μM) and TSA (0.1 μM). **D** Dose-dependent effects of chidamide on cell proliferation over time in sensitive (KHYG1 and MEC04) and resistant (HANK1 and SNK6) cell lines. **E** Cell cycle assay of KHYG1, MEC04, HANK1 and SNT8 cells treated with chidamide for 72 h. **F** Annexin V/PI staining of KHYG1 and HANK1 cells treated with chidamide for 72 h. The results are expressed as the mean ± SD of three independent experiments

To assess the in vitro efficacy of chidamide in NKTL, we evaluated the IC50 of the drug in 11 NKTL cell lines (Fig. 2A) and divided them into chidamide-sensitive and chidamide-resistant groups. We further confirmed our observations by exposing these cell lines to 1 μM chidamide and found that the KHYG1, MEC04 and NK92 cell lines were sensitive, while the HANK1, SNK6 and SNT8 cell lines were resistant to the cytotoxic effect of the drug (Fig. 2B). Similar observations were also obtained with other HDAC inhibitors, such as romidepsin, belinostat, SAHA and TSA (Fig. 2C). Moreover, a dose-response study showed that chidamide could effectively suppress the proliferation of KHYG1 and MEC04 cells in a dose-dependent manner but did not have this effect in the chidamide-resistant cell lines HANK1 and SNK6 (Fig. 2D). We further showed that chidamide most likely induced cell death through apoptosis, as demonstrated by the increased Annexin V/PI staining in treated cells (Fig. 2E, F). Collectively, these results suggest that some NKTL cell lines are sensitive to chidamide, while others are resistant to the drug, recapitulating the clinical trial results.

### Enhancer landscapes in chidamide-resistant and chidamide-sensitive cells

**Fig. 3:**
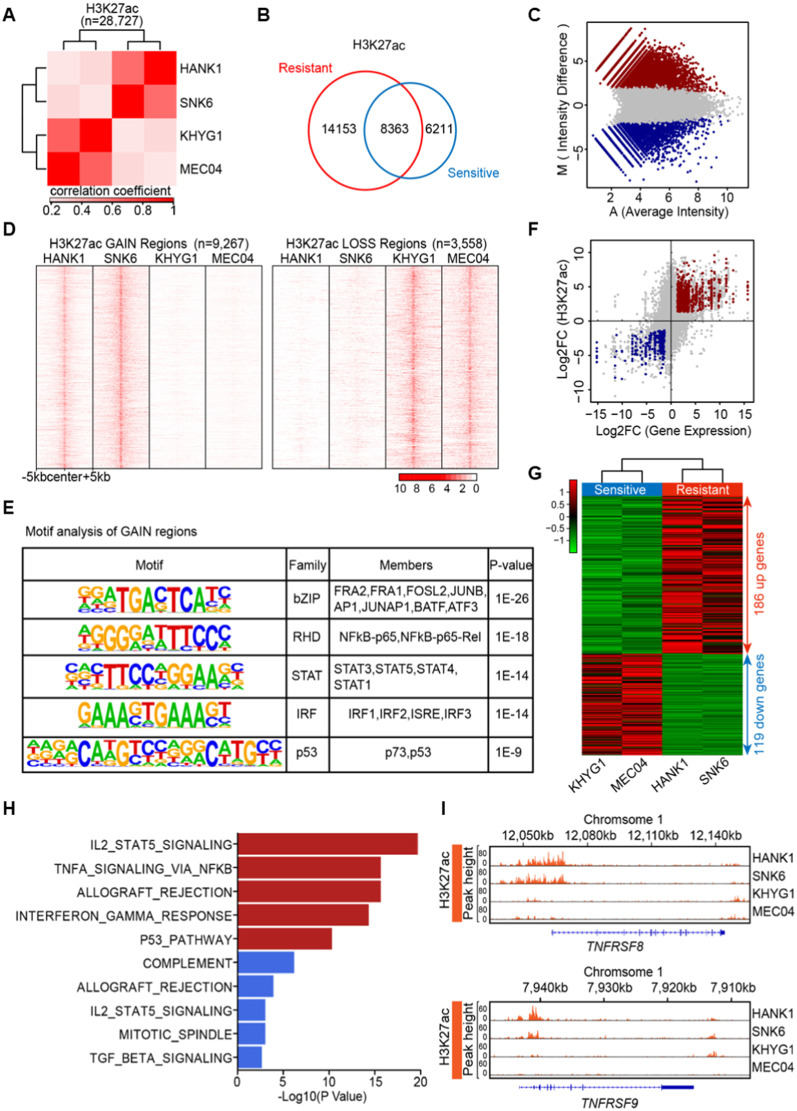
Chromatin and transcriptomic profiling of chidamide-resistant and chidamide-sensitive NKTL cells. **A** Heatmap representation of unsupervised hierarchical clustering based on H3K27ac occupancy at total H3K27ac ChIP-seq peaks (*n* = 28,727). Samples were clustered based on the Spearman correlation coefficient with average linkage. **B** Venn diagram showing the overlap of H3K27ac peaks between resistant and sensitive cells. **C** MA plot of all peaks from comparison of resistant and sensitive samples after normalization by MAnorm3. Each dot represents a peak, the red dots represent the GAIN regions (*n* = 9267), and the blue dots represent the LOSS regions (*n* = 3558). **D** Heatmap representation of GAIN and LOSS regions based on H3K27ac occupancy in resistant and sensitive samples. Ten kb around the center of the GAIN and LOSS regions is displayed for each sample. **E** Motif analysis of GAIN regions in resistant cells using HOMER, showing significant enrichment of the bZIP family, RHD family, STAT family and IRF family (hypergeometric test). **F** Scatter plot showing the correlation between ChIP-seq mark intensity and RNA-seq read intensity. Each dot represents an overlapping locus, while the red dots (*n* = 906) indicate upregulated loci between resistant and sensitive cells, and the blue dots (*n* = 340) indicate downregulated loci. **G** Unsupervised hierarchical clustering of differentially expressed genes (DEGs) (*p* < 0.05, |log2-fold change|> 1) overlapping with H3K27ac GAIN and LOSS regions in sensitive versus resistant cells. **H** GSEA was performed based on a hypergeometric test that takes the size of the overlap between the hallmark gene set and the list of differentially expressed genes overlapping with H3K27ac GAIN and LOSS regions in resistant versus sensitive cells as the test statistics. **I** IGV screen shots showing the distribution of H3K27ac mark intensity in resistant versus sensitive cells at two representative genomic loci (*TNFRSF8* and *TNFRSF9*)

To investigate the underlying mechanism of resistance to chidamide in NKTL cells, we performed chromatin immunoprecipitation sequencing (ChIP-seq) for H3K27ac and H3K4me3 in two resistant cell lines (HANK1 and SNK6) and two sensitive cell lines (KHYG1 and MEC04). Epigenetic changes in H3K27ac and H3K4me3, which are associated with gene enhancers and promoters, respectively, have been shown to modulate gene activities and confer resistance to drug treatment [23, 24]. Unsupervised clustering of H3K27ac peaks (*n* = 28,727) revealed that the H3K27ac signals were significantly different between resistant and sensitive cells (Fig. 3A), whereas no significant difference in H3K4me3 peaks (*n* = 19,615) was found (Additional File 1: Fig. S1), suggesting that enhancer alterations rather than promoter changes may play an important role in mediating chidamide resistance. We compared the H3K27ac peaks between the sensitive and resistant cells and found that 8363 peaks were common to both, and 14,153 peaks and 6211 peaks were unique to resistant and sensitive cells, respectively (Fig. 3B). The quantitative binding differences inferred by MAnorm3 showed that most H3K27ac peaks in resistant cells were significantly different from the peaks in sensitive cells, including 9267 regions with increased H3K27ac (GAIN regions) and 3558 regions with decreased H3K27ac (LOSS regions) in resistant cells (Fig. 3C, Additional File 2: Table S5). We observed that while the density of ChIP-seq reads for H3K27ac showed consistency within the chidamide-resistant cell lines (HANK1 and SNK6) and within the chidamide-sensitive cell lines (KHYG1 and MEC04), there were remarkable differences between the resistant and sensitive groups, suggesting that differential enhancer marks might play an important role in determining the response to chidamide (Fig. 3D). Furthermore, motif analysis of GAIN regions in the resistant cell lines using HOMER revealed that the top enriched families were the bZIP family, RHD family, STAT family and IRF family (Fig. 3E, Additional File 2: Table S6) [25], suggesting that transcription factors in these families, such as NFkB and STAT, might mediate resistance-dependent chromatin remodeling at gained enhancers.

To gain further insights into the altered transcriptional pathways resulting from epigenetic dysregulation, RNA-seq was performed in two chidamide-resistant (HANK1 and SNK6) and two chidamide-sensitive (KHYG1 and MEC04) cell lines. Integrative transcriptomic and chromatin profiling analyses revealed that the differential H3K27ac peaks were positively correlated with gene expression in NKTL cells (Fig. 3F, Additional File 2: Table S7). In total, 186 upregulated genes and 119 downregulated genes were identified from integrative analysis of both RNA-seq and H3K27ac changes in resistant cells when compared with sensitive cells (Fig. 3G, Additional File 2: Table S8). Gene set enrichment analysis (GSEA) using the hallmark gene set and based on a hypergeometric distribution of the differentially expressed genes (DEGs) between resistant and sensitive cells revealed that the JAK-STAT pathway was one of the most upregulated pathways in chidamide-resistant cells, suggesting that this pathway has a significant role in NKTL resistance to chidamide (Fig. 3H). We investigated the ChIP-seq profiles of two typical JAK-STAT target genes, *TNFRSF8* and *TNFRSF9*. Increased H3K27ac signal intensities at the enhancers of the *TNFRSF8* and *TNFRSF9* gene loci were observed in HANK1 and SNK6, but not in KHYG1 and MEC04 cells (Fig. 3I). Together, these results suggest that chromatin remodeling associated with resistance to chidamide in NKTL may be mediated by aberrant JAK-STAT activity.

### Aberrant activation of the JAK-STAT pathway in NKTL

**Fig. 4:**
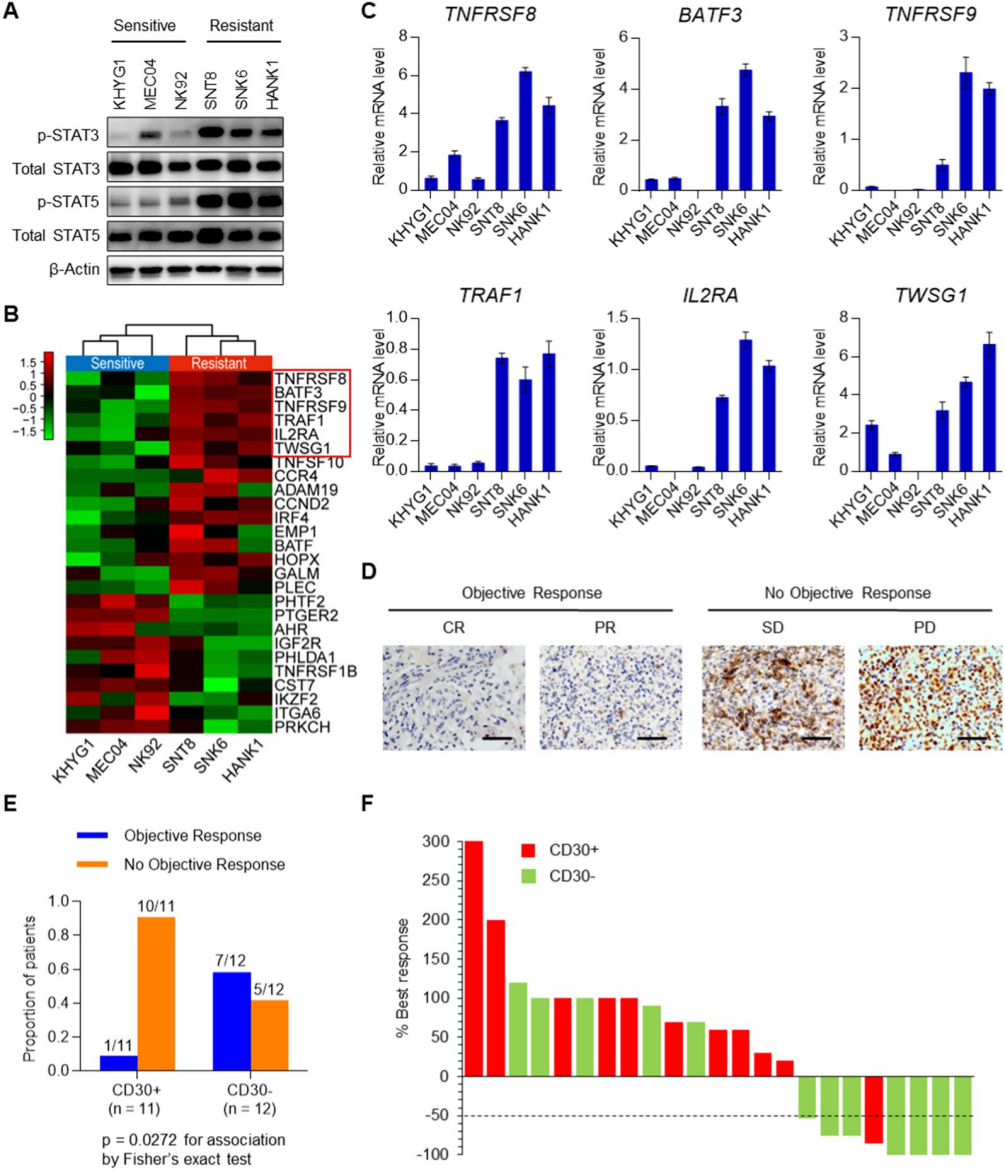
The JAK-STAT pathway is constitutively activated in chidamide-resistant NKTL. **A** The JAK-STAT oncogenic signaling pathway was activated in chidamide-resistant cells. **B** Hierarchical clustering of DEGs from the JAK-STAT pathway in sensitive versus resistant cells. **C** RT–qPCR validation of genes from the JAK-STAT pathway in sensitive and resistant cells. **D** Representative images of IHC for CD30 in NKTL biopsies. Scale bars: 50 μm. **E** Association between the expression of CD30 and clinical response. The expression of CD30 in 23 NKTL biopsies (available from the 28 relapsed/refractory NKTL patients) was examined by IHC. *p* = 0.0272 for association by Fisher’s exact test. **F** Waterfall plot showing the association between the expression of CD30 and the best overall response to chidamide in 23 patients. The results are expressed as the mean ± SD of three independent experiments

As the JAK-STAT pathway was found to be implicated in resistance to chidamide, we examined the activity of its effector molecules, STAT3 and STAT5 in both sensitive and resistant NKTL cell lines. High levels of phospho-STAT3 and STAT5 were detected in resistant cell lines compared to sensitive cell lines (Fig. 4A). Aberrant STAT3 activity associated with the presence of activating mutations in the STAT3 gene [21] was also observed in chidamide-resistant cell lines in this study, suggesting that hyperactivation of the JAK-STAT pathway confers chidamide resistance in NKTL. All three chidamide-resistant cell lines, HANK1 (G618R), SNK6 (D661Y) and SNT8 (D661Y), harbored *STAT3* activating mutations, which were not found in the three chidamide-sensitive cell lines (KHYG1, NK92 and MEC04) [26]. Upregulated JAK-STAT target genes in NKTL were verified with transcriptomic data and RT–qPCR (Fig. 4B, C, Additional File 2: Table S9). Since TNFRSF8 (CD30) was found to be a downstream target is of the JAK-STAT pathway, we hypothesized that CD30 expression would be associated with resistance to chidamide treatment. We performed immunohistochemistry (IHC) for CD30 with available NKTL biopsies (from the 28 relapsed/refractory NKTL patients). CD30 expression was detected in 48% (11/23) of NKTL patients, and of these patients, 91% (10/11) were resistant to chidamide treatment, showing that CD30 expression was significantly associated with inferior responses to chidamide in NKTL (*p* = 0.0272, Fig. 4D–F). Collectively, these findings suggest that the expression of JAK-STAT target genes, such as CD30 (TNFRSF8), could be a potential predictive biomarker for chidamide resistance in NKTL.

### Pharmacologically targeting the JAK-STAT pathway overcomes resistance to chidamide in NKTL

**Fig. 5:**
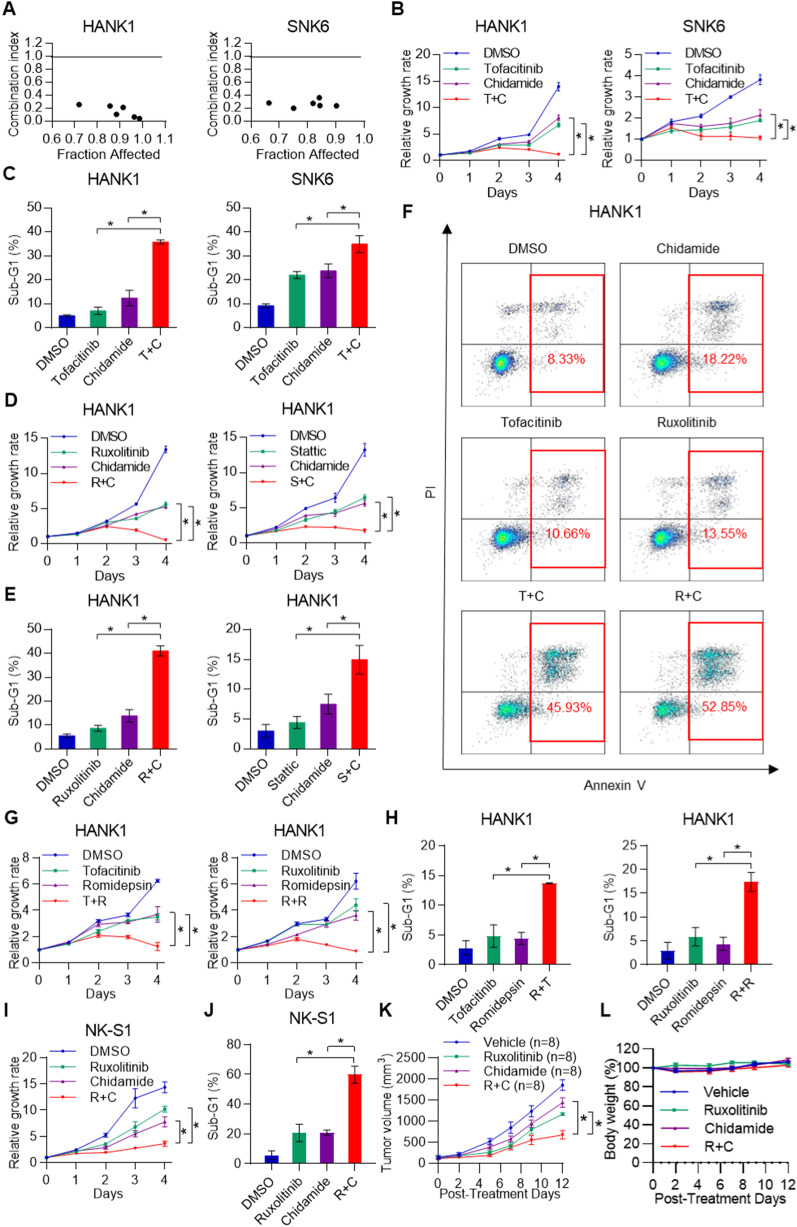
JAK-STAT inhibitors overcome chidamide resistance in NKTL cells. **A** Combination index values for chidamide and tofacitinib combinatorial treatment in HANK1 and SNK6 cells. Combination index values were calculated with CalcuSyn software as a function of the level of antiproliferative activity. Combination index = 1 denotes additivity, combination index > 1 denotes antagonism, and combination index < 1 denotes synergy. **B** Proliferation curves in the presence of tofacitinib, chidamide or a combination. **C** Cell cycle assay for the combined effects of tofacitinib and chidamide in HANK1 and SNK6 cells. **D–E** Proliferation curves and cell cycle assays for combination treatment with other JAK-STAT inhibitors, ruxolitinib/Stattic and chidamide, in HANK1 cells. **F** Annexin V/PI staining of HANK1 cells treated with chidamide and tofacitinib/ruxolitinib for 72 h. **G** Proliferation curves in the presence of tofacitinib/ruxolitinib, romidepsin or a combination in HANK1 cells. **H** Cell cycle assay for the combination treatment of HANK1 cells with romidepsin and tofacitinib/ruxolitinib for 72 h. **I** Proliferation curve of NK-S1 cells in the presence of ruxolitinib, chidamide or a combination. **J** Cell cycle assay for the combination treatment of NK-S1 cells with ruxolitinib and chidamide for 72 h. **K** Xenograft tumor growth curve of NK-S1 cells in NOD/SCID/IL2rγnull (NSG) mice treated with chidamide at 25 mg/kg, ruxolitinib at 180 mg/kg or both for 12 days. **L** Body weight of NSG mice bearing NK-S1 tumors treated with chidamide at 25 mg/kg, ruxolitinib at 180 mg/kg or both for 12 days. The results are expressed as the mean ± SD of three independent experiments. *p < 0.05

We hypothesized that targeting the JAK-STAT pathway could reverse chidamide resistance in NKTL cell lines. To test this hypothesis, we treated the chidamide-resistant NKTL cell lines (HANK1 and SNK6) with tofacitinib (JAK inhibitor), chidamide or a combination of both drugs for 96 h and determined the combination index (Fig. 5A). Combination treatment of tofacitinib and chidamide in NKTL-resistant cell lines synergistically inhibited their proliferation compared with single-drug treatment (Fig. 5B). In addition, the combination treatment significantly induced cell death compared to the single-drug treatment (Fig. 5C), suggesting that JAK inhibition could overcome chidamide resistance in NKTL. Similar results were observed when another JAK inhibitor (ruxolitinib) or a STAT inhibitor (Stattic) was tested in combination with chidamide (Fig. 5D, E), indicating that blockade of the JAK-STAT pathway in general may overcome chidamide resistance, probably through induction of apoptosis (Fig. 5F). Similarly, we proceeded to determine whether JAK-STAT inhibition could overcome resistance to other HDAC inhibitors, such as romidepsin, SAHA and TSA. Consistently, combination treatment with tofacitinib/ruxolitinib and romidepsin significantly blocked cell proliferation and induced cell death in HANK1 cells compared to single-drug treatment (Fig. 5G, H). Tofacitinib/ruxolitinib in combination with SAHA/TSA also significantly induced cell death and apoptosis in HANK1 cells (Additional File 1: Fig. S2A–C). Collectively, these results indicate that combined inhibition of JAK-STAT signaling and HDACs leads to the efficient killing of NKTL cells due to the synergistic activation of apoptosis. To further demonstrate the combined effect of chidamide and a JAK inhibitor in vivo, we applied the combined treatment in our NK-S1 xenograft model. The two chidamide-resistant NKTL cell lines (HANK1 and SNK6) do not form xenografts in NSG mice, making them unsuitable for in vivo study, as both cell lines are dependent on the presence of recombinant IL2 for cell proliferation. Thus, the NK-S1 cell line, which is partially resistant to chidamide and could readily form xenografts, was selected for this study. Combination treatment with chidamide and ruxolitinib significantly inhibited cell proliferation and induced cell death in NK-S1 cells in vitro (Fig. 5I, J), similar to that seen in HANK1 cells (Fig. 5D, E). In the NK-S1 xenograft model, chidamide in combination with ruxolitinib retarded tumor growth compared with chidamide or ruxolitinib alone (Fig. 5K). In addition, chidamide, ruxolitinib and their combination were well tolerated, as demonstrated by the maintenance of body weight in the treatment group (Fig. 5L). Together, these preclinical data support the use of chidamide in combination with ruxolitinib as a promising strategy in relapsed/refractory NKTL patients.

### The JAK inhibitors tofacitinib/ruxolitinib induce chromatin remodeling at JAK-STAT genes

**Fig. 6:**
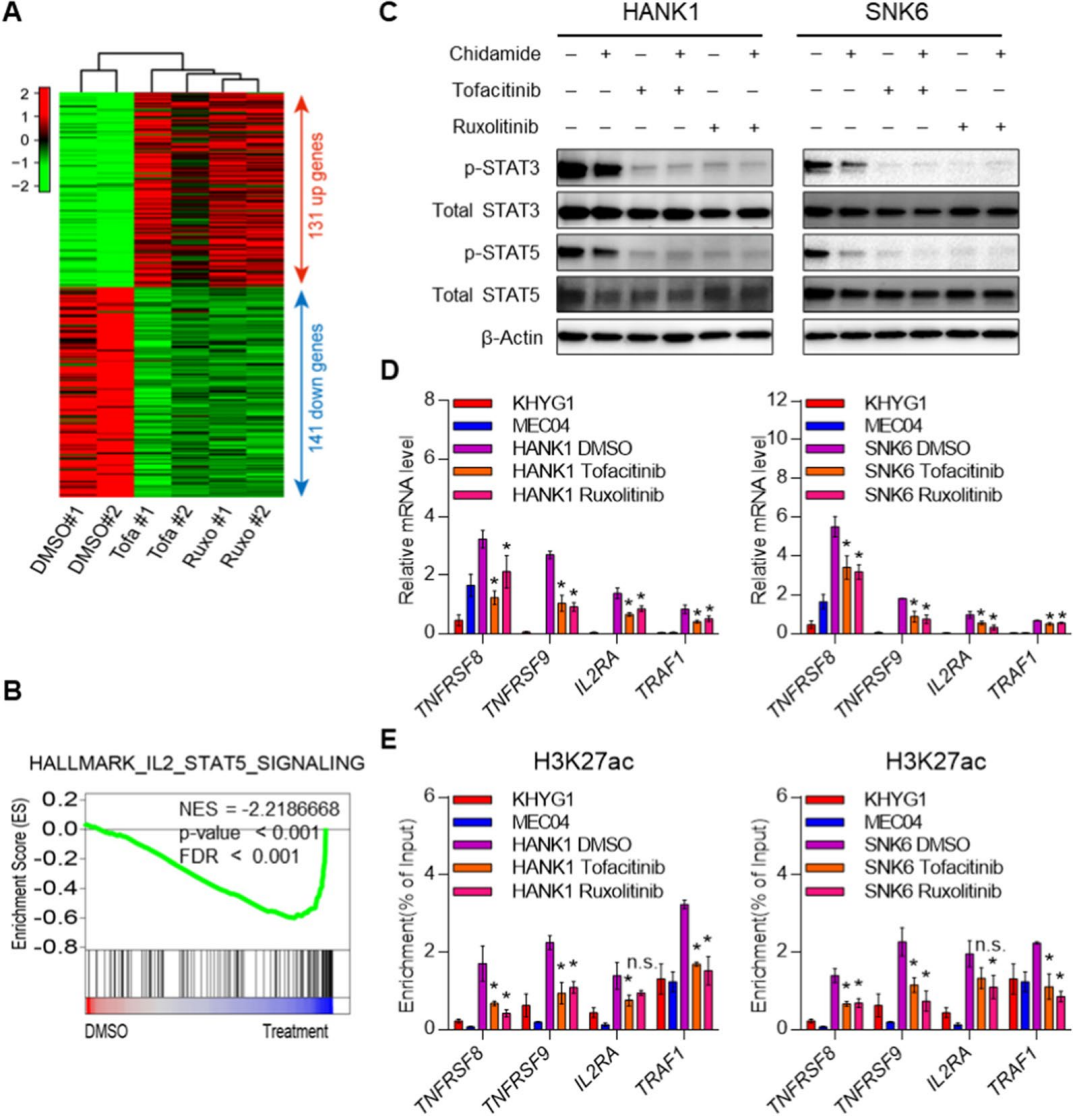
The JAK inhibitors tofacitinib and ruxolitinib reverse chidamide resistance by altering chromatin remodeling. **A** Hierarchical clustering showing the DEGs in HANK1 cells treated with tofacitinib or ruxolitinib versus DMSO (*p* < 0.05, |log2-fold change|> 1). **B** GSEA pathways analysis of the JAK-STAT pathway after tofacitinib or ruxolitinib treatment versus DMSO treatment of HANK1 cells. **C** The JAK inhibitors tofacitinib and ruxolitinib impaired the JAK-STAT pathway in resistant NKTL. **D** RT–qPCR validation of genes in the JAK-STAT pathway for sensitive cells and resistant cells with or without tofacitinib/ruxolitinib treatment. **E** ChIP-qPCR of genes in the JAK-STAT pathway for sensitive cells and resistant cells with or without tofacitinib/ruxolitinib treatment. HANK1/SNK6 cells were treated with tofacitinib/ruxolitinib or DMSO. The results are expressed as the mean ± SD of three independent experiments. *p < 0.05, n.s., not significant

To understand how JAK inhibitors reverse chidamide resistance in NKTL cells, we performed RNA-seq of HANK1 cells treated with DMSO, tofacitinib or ruxolitinib. Compared to the control treatment, tofacitinib and ruxolitinib treatment remarkably upregulated 131 common genes and downregulated 141 common genes in HANK1 cells (Fig. 6A, Additional File 2: Table S10). GSEA revealed that most genes in the JAK-STAT pathway were downregulated in HANK1 cells treated with tofacitinib or ruxolitinib (Fig. 6B). Tofacitinib/ruxolitinib alone or in combination with chidamide effectively downregulated the JAK-STAT pathway and its downstream target genes in chidamide-resistant NKTL (Fig. 6C, D), suggesting that these inhibitors could induce JAK-STAT pathway reprogramming. To investigate whether this reprogramming is due to chromatin remodeling, we performed ChIP-qPCR for H3K27ac in sensitive cells and resistant cells with or without tofacitinib/ruxolitinib treatment and examined the H3K27ac changes of typical genes in the JAK-STAT pathway, such as TNFRSF8, TNFRSF9, IL2RA and TRAF1. We found that the ChIP signal (compared to percent input) of these genes in resistant cells was much higher than that in sensitive cells and was reduced after tofacitinib/ruxolitinib treatment (Fig. 6E). These results suggest that the chromatin accessibility of chidamide-resistant cells was reversed to a state inducing sensitivity of cells after blockade of the JAK-STAT pathway by tofacitinib/ruxolitinib treatment.

### Clinical efficacy of JAK-STAT inhibition and chidamide in chidamide-resistant NKTL

**Fig. 7:**
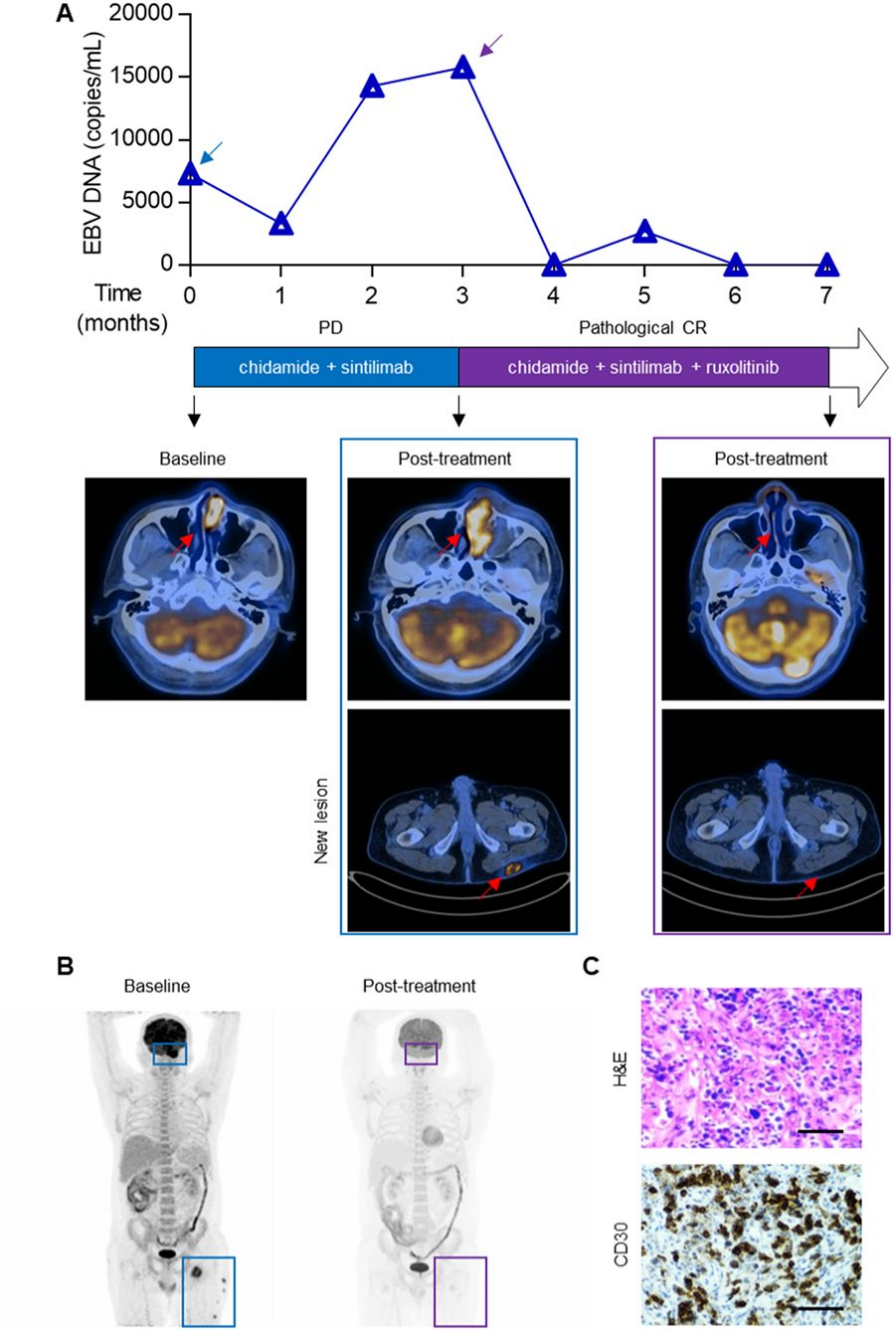
The benefit of combinatorial therapy in relapsed/refractory NKTL patient. **A** Top panel, graph depicting the dynamics of EBV DNA copy number in the blood of a relapsed/refractory NKTL patient during treatment with a series of combinatorial therapies. The blue and purple arrows indicate the start time of treatment with the combination of chidamide + sintilimab and chidamide + sintilimab + ruxolitinib, respectively. Middle panel, clinical response to the indicated therapeutic regimens. Bottom panel, representative scans from the patient at baseline and after treatment with the combination of chidamide + sintilimab or chidamide + sintilimab + ruxolitinib. The red arrows indicate lesion locations. **B** Whole-body PET-CT showing tumor lesions (blue and purple frames) before and after combination treatment with chidamide + sintilimab + ruxolitinib in a relapsed/refractory NKTL patient. **C** Representative images of H&E staining (upper) and IHC for CD30 (bottom) in an NKTL biopsy before combination treatment with chidamide + sintilimab + ruxolitinib. Scale bars, 50 μm

We next assessed whether JAK-STAT inhibition could be a potential therapeutic strategy to overcome resistance to chidamide in the clinic. A 48-year-old Chinese male patient who was initially diagnosed with stage IIA extranodal NKTL of the nasal type was recruited. He was refractory to first-line asparaginase-based chemotherapy. Salvage therapy with a combination of chidamide (30 mg orally, twice a week, continuous) and the anti-programmed death protein 1 (PD-1) antibody sintilimab (200 mg intravenously, on Day 1) was given, but the disease progressed with multiple new lesions accompanied by a dramatic increase in the peripheral blood Epstein–Barr virus (EBV) viral load after four 21-day cycles (Fig. 7A). The JAK inhibitor ruxolitinib was added (10 mg orally, twice a day) to his therapeutic regimen, and the patient achieved PR after 2 cycles of the combination treatment (chidamide + sintilimab + ruxolitinib). After an additional 2 cycles of treatment, the patient achieved CR evaluated by PET-CT (Fig. 7A, B) and experienced a disease-free survival time of more than 22 months as of the last follow-up in July 2021. Recent studies have reported that inactivating mutations of JAK1/2 are associated with resistance to PD-1 blockade in melanoma [27–29], whereas genetic inhibition of the JAK-STAT pathway results in resistance to anti-PD-1 therapy [30], suggesting that the complete response to our salvage therapy was mainly due to the combined effects of ruxolitinib and chidamide but not PD-1 blockade. A high level of CD30 expression was detected in the pretreatment biopsy from the patient, supporting the potential use of CD30 as a biomarker for patient stratification for chidamide + ruxolitinib combination therapy (Fig. 7C). Collectively, combination treatment with chidamide and JAK-STAT inhibitors could be a novel option for treating relapsed/refractory NKTL patients.

## Discussion

In the current management of NKTL, a substantial proportion of patients still experience treatment failures, and salvage options are severely limited. Increasing evidence suggests that PTCL is characteristically an epigenetic disorder, with several HDAC inhibitors receiving regulatory approvals for the treatment of relapsed/refractory disease. Here, we conducted the first focused trial of chidamide, an oral HDAC inhibitor, in relapsed/refractory NKTL. Chidamide induced durable tumor regression, with a complete response rate of 18% and a partial response rate of 21%. Compared with other HDAC inhibitors, chidamide is the first selective oral HDAC inhibitor to show effective and increased immunomodulatory effects and convenient administration [31, 32]. Although HDAC inhibitors have been approved for relapsed/refractory T cell lymphomas, including PTCL and CTCL [33], this is the first successful application of the HDAC inhibitor chidamide in a focused study of relapsed/refractory NKTL in which the underlying drug resistance mechanisms in this disease have been investigated and reported.

We further found that aberrant JAK-STAT signaling contributes to primary resistance to chidamide, and inhibiting this oncogenic pathway can resensitize resistant NKTL cells to chidamide. We have previously demonstrated the prevalence of JAK-STAT pathway alterations in NKTL and discovered frequent *STAT3* activating mutations in 21% of patients with this disease [21]. All three chidamide-resistant cell lines featured in Fig. 4A (HANK1, SNK6 and SNT8) harbored *STAT3* activating mutations, which were not found in the three chidamide-sensitive cell lines (KHYG1, NK92 and MEC04), suggesting that mutations might be one of the underlying mechanisms leading to activation of the JAK/STAT pathway in NKTL, which confers resistance to chidamide. Our study shows that hyperactivation of JAK-STAT signaling confers resistance to chidamide in NKTL, suggesting that the activity of JAK-STAT may serve as a predictive biomarker of the response to chidamide.

Interestingly, we found that JAK-STAT signaling mediates resistance to chidamide through chromatin remodeling of enhancer elements. We demonstrated that drug resistance to chidamide was correlated with chromatin remodeling alterations, which could be reversed by targeting the JAK-STAT pathway. Enhancer but not promoter alterations were significantly correlated with the response to chidamide in NKTL. In addition, STAT motifs were enriched at gained enhancers over lost enhancers, which was consistent with the enrichment of JAK-STAT target genes in resistant cells. Previous studies have demonstrated that the JAK-STAT pathway can regulate chromatin accessibility and influence the chromatin landscape through different mechanisms. For example, JAK2 can directly phosphorylate Tyr41 in histone H3 (H3Y41), preventing the binding of heterochromatin protein 1α (HP1α) with chromatin and destabilizing heterochromatin in hematopoietic cells [34]. The carboxy-terminal transactivation domains (TADs) of STAT recruit coactivator HATs, such as CREBBP/p300, to reprogram chromatin [35]. STAT3 can also regulate H3K4me3 of its target genes in T cells under Th17 cell-polarizing conditions and recruit DNA methyltransferase 1 (DNMT1) to gene promoters to silence tumor suppressor genes, such as SHP-1, in malignant T lymphocytes [36–38]. In myeloproliferative neoplasms (MPNs), a recent study showed that constitutive JAK2 activation induces chromatin-level alterations, particularly in enhancer utilization which promotes NFkB signaling, resulting in resistance to JAK inhibition [39]. Therefore, our study may provide potential clinical opportunities that can be exploited to overcome resistance to HDAC inhibitors.

Importantly, chromatin-mediated drug resistance is often reversible, as demonstrated by JAK-STAT inhibitor reprogramming of the chromatin state from a resistant to a sensitive state. JAK-STAT inhibition suppresses H3K27ac marks of JAK-STAT target genes, such as *TNFRSF8* and *TNFRSF9*. Consequently, inhibition of JAK-STAT overcomes resistance to chidamide. A recent study showed that massive enhancer remodeling plays a key role in acquired resistance to anaplastic lymphoma kinase (ALK) inhibitors and that panobinostat can induce chromatin remodeling in resistant cells to overcome acquired resistance to ALK inhibitors in ALK-positive lung cancer [40]. Knoechel et al. demonstrated that T-cell acute lymphoblastic leukemia (T-ALL) can acquire resistance to γ-secretase inhibitors (GSIs) via a fully reversible epigenetic mechanism, and GSIs and the BRD4 inhibitor JQ1 have synergistic effects on GSI-tolerant “persister” cells [41]. Moreover, Zawistowski et al. found that the MEK inhibitor trametinib induces enhancer remodeling during adaptive resistance and that JQ1 can overcome this resistance by impairing enhancer remodeling in triple-negative breast cancer (TNBC) [42]. Consistently, our study indicates that targeting chromatin alterations such as gained enhancers is beneficial in chidamide-resistant NKTL patients.

Furthermore, JAK-STAT target genes can also serve as predictive or therapeutic biomarkers of chidamide resistance. Frequent overexpression of TNFRSF8 (CD30) in NKTL has been reported previously [43, 44]. CD30 expression in NKTL was significantly associated with better clinical responses when patients were treated with non-anthracycline-based chemotherapy [45]. In this study, we observed that CD30 was overexpressed in both chidamide-resistant cell lines and tumor samples from patients demonstrating primary resistance to chidamide. Potentially, our discovery may provide the basis for the use of brentuximab vedotin (an anti-CD30 antibody–drug conjugate), which has recently been used in a successful phase III trial for patients with CD30-positive peripheral T-cell lymphomas, in this subgroup of NKTL patients to improve survival [46].

## Conclusions

In summary, our study shows favorable clinical outcomes of chidamide in treating relapsed/refractory NKTL patients. JAK-STAT inhibitors may further expand the clinical utility of HDAC inhibitors, including chidamide, romidepsin and belinostat, by remodeling the resistant enhancer landscape toward a sensitive state. The combined use of JAK-STAT and HDAC inhibitors is a promising new treatment option for relapsed/refractory NKTL patients. We believe that our study could promote the clinical application of HDAC inhibitor chidamide in NKTL, provide personalized and precision treatments for NKTL patients and improve their survival rate.

## Supplementary Information

**Additional file 1**. **Supplementary Methods and Figures S1–2. Figure S1**. H3K4me3 landscapes in chidamide-resistant and chidamide-sensitive NKTL.** Figure S2**. JAK inhibitors overcome the resistance of SAHA/TSA in NKTL cells.

**Additional file 2**. **Tables S1-10**.** Table S1**. List of primers used for ChIP-qPCR.** Table S2**. List of primers used for RT–qPCR.** Table S3**. Demographic and clinical characteristics of the 28 relapsed/refractory NKTL patients.** Table S4**. Patient characteristics at baseline.** Table S5**. List of GAIN and LOSS regions identified in both resistant and sensitive samples.** Table S6**. List of motifs in GAIN regions.** Table S7**. List of GAIN and LOSS regions overlapping with nearest genes identified in both resistant and sensitive samples.** Table S8**. List of differentially expressed genes overlapping with GAIN and LOSS regions in sensitive versus resistant samples.** Table S9**. Differentially expressed genes from JAK-STAT pathway in sensitive versus resistant samples.** Table S10**. Differentially expressed genes in HANK1 treated with DMSO, tofacitinib or ruxolitinib.

## Data Availability

The datasets supporting the conclusions of this article are available at GEO under accession number GSE152185.

## Abbreviations

ALK: Anaplastic lymphoma kinase
ASCT: Autologous stem cell transplantation
ATL: Adult T-cell leukemia
ChIP-Seq: Chromatin immunoprecipitation sequencing
CI: Combination index
CR: Complete response
CTCL: Cutaneous T-cell lymphoma
DEGs: Differentially expressed genes
DLBCL: Diffuse large B-cell lymphoma
DNMT1: DNA methyltransferase 1
EBER ISH: Epstein–Barr encoding region in situ hybridization
EBV: Epstein–Barr virus
FFPE: Formalin-fixed, paraffin-embedded
FK-228: Romidepsin
GSEA: Gene set enrichment analysis
GSI: γ-Secretase inhibitor
HATs: Histone acetyltransferases
HDAC: Histone deacetylase
HDACs: Histone deacetylases
HP1α: Heterochromatin protein 1 α
H3Y41: Tyr41 on histone H3
IC50: Half maximal inhibitory concentration
IHC: Immunohistochemistry
MPN: Myeloproliferative neoplasms
NHL: Non-Hodgkin's lymphoma
NKTL: Natural killer/T-cell lymphoma
NMPA: National Medical Products Administration
NSG: NOD/SCID/IL2rγnull
ORR: Objective response rate
OS: Overall survival
PD: Progressive disease
PD-1: Programmed death protein 1
PET-CT: Positron emission tomography-computed tomography
PFS: Progression-free survival
PI: Propidium iodide
PMDA: Pharmaceuticals and Medical Devices Agency
PR: Partial response
PTCL: Peripheral T-cell lymphoma
RNase A: Ribonuclease A
SAHA: Vorinostat
SD: Stable disease
SYSUCC: Sun Yat-sen University Cancer Center
TADs: Transactivation domains
TCL: T-cell lymphoma
TNBC: Triple-negative breast cancer
TSA: Trichostatin A
T-ALL: T-cell acute lymphoblastic leukemia
