# Supplementary material for: Aberrant JAK-STAT signaling-mediated chromatin remodeling impairs the sensitivity of NK/T-cell lymphoma to chidamide
Source: Clin Epigenetics. 2023 Feb 6;15:19. doi: 10.1186/s13148-023-01436-6 (PMC9900953; doi:10.1186/s13148-023-01436-6)
Supplement: Supplementary file 1 — Additional file 1. Supplementary Methods and Figures S1–2. Figure S1. H3K4me3 landscapes in chidamide-resistant and chidamide-sensitive NKTL. Figure S2. JAK inhibitors overcome the resistance of SAHA/TSA in NKTL cells. [file 13148_2023_1436_MOESM1_ESM.pdf]

INDEX OF SUPPLEMENTARY DATA

Supplementary Methods

Supplementary References

Supplementary Figures

Supplementary Methods

Patients and cell lines

Refractory was defined as not achieving partial response (PR) or complete response (CR) after at least two cycles of asparaginase based chemotherapy according to evaluation criteria of malignant lymphoma (Lugano 2014 Criteria). Patients aged 18 to 75 years with diagnosed relapsed/refractory NKTL after prior asparaginase-based chemotherapy or chemoradiotherapy were eligible if they had ECOG performance status ≤ 2. Adequate organ and bone marrow function, no severe hematopoietic dysfunction, cardiac, pulmonary, liver, kidney, thyroid dysfunction and at least one measurable or evaluable lesion. Patients with central nervous system involvement or hemophagocytic syndrome were excluded from the trial. All patients did not receive any systemic antineoplastic therapy within 28 days before chidamide treatment, and they were treated with 30 mg chidamide twice a week (BIW) or 10 mg chidamide per day (QD) orally until disease progression or intolerance. The primary endpoint was objective response rate (ORR) assessed by investigators according to the Lugano 2014 criteria. Key secondary endpoints included time to response, duration of response and progression-free survival, overall survival and safety.

HANK1 was a generous gift from Dr. Yoshitoyo Kagami and YT was from Dr. C. Clayberger. SNK6, SNT8, SNK1 and NKYS were kindly provided by Dr. Norio Shimizu. MEC04 was a gift from Dr. Paul Coppo and Dr. Philippe Gaulard. KHYG1

and KAI-3 were obtained from the Japanese Collection of Research BioResources (JCRB) cell bank. NK92 was purchased from American Type Culture Collection (ATCC). NK-S1 was established from a NKTL xenograft [1].

HANK1 and SNT8 were cultured in Artemis medium-2 (ScyMed Inc.) supplemented with 2% human plasma serum (Clontech) and 1% penicillin-streptomycin (P/S, Thermo Fisher Scientific). 100 IU/ml interleukin 2 (IL-2, Miltenyi Biotec) was included into HANK1 growth media and 700 IU/ml IL-2 into SNT8, respectively. SNK1, SNK6 and NK92 were maintained in RPMI1640 medium (Thermo Fisher Scientific) supplemented with 10% FBS (Hyclone), 10% horse serum (Thermo Fisher Scientific), 1% P/S and 100 IU/ml IL-2. KHYG1, KAI-3 and NKSY were grown in RPMI1640 medium supplemented with 10% FBS, 1% P/S and 100 IU/ml IL-2. MEC04 was cultured in RPMI1640 medium supplemented with 15% FBS, 1% P/S and 50 IU/ml IL-2. NK-S1 was maintained in DMEM medium (Thermo Fisher Scientific) supplemented with 10% FBS, 10% horse serum and 1% P/S. YT was grown in IMDM medium (Thermo Fisher Scientific) supplemented with 20% FBS, 1% Sodium pyruvate (Thermo Fisher Scientific) and 1% P/S. All cultures were routinely checked for mycoplasma contamination.

Western blot

A total of 5 x 10^5 cells were treated with or without chidamide and tofacitinib/ruxolitinib for 24 hours and harvested for protein extraction. The primary

antibodies are purchased from Cell Signaling Technology: pSTAT3 (#9145s), STAT3 (#9139s), pSTAT5 (#9356s), STAT5 (#9363s) and β-actin (#8457s). The secondary antibodies used are ECL Rabbit IgG, HRP-linked whole Ab (from donkey) (GE Healthcare, #NA934-1ML) and ECL Mouse IgG, HRP-linked whole Ab (from sheep) (GE Healthcare, #NA931-1ML). The signals were detected using Tanon™ High-sig ECL Western Blotting Substrate (Tanon).

ChIP-qPCR, ChIP-seq and ChIP-seq data analyses

Briefly, a total of 1 x 10^6 cells were fixed in 1% formaldehyde for 10 minutes at room temperature. Fixation was stopped by adding glycine to 0.125 M. Cells were then washed 3 times with TBSE buffer, lysed in 100 μl SDS lysis buffer and sonicated for 16 cycles (30 seconds on, 30 seconds off) using Bioruptor (Diagenode). The chromatin solution was precleared with protein G Dynabeads (Thermo Fisher Scientific) for 1 hour at 4°C and incubated with antibody-beads complex in cold room overnight for immunoprecipitation. The amount of antibody H3K27ac (Abcam) and H3K4me3 (Millipore) used was 2 μg. Next day, the complexes were washed for a total of 4 washings, eluted and reverse crosslinked using Pronase A (Roche) with incubation at 42°C for 2 hours and 68°C for 6 hours. The reverse crosslinked DNA were purified by Phenol-CHCl3-Isoamy alcohol (Thermo Fisher Scientific) and precipitated by ethanol. ChIP-qPCR was performed using SYBR™ Green PCR Master Mix (Thermo Fisher Scientific). Enrichment data were analyzed by calculating the immunoprecipitated DNA as a percentage of input DNA. For ChIP-Seq, ChIP

DNA and input DNA were amplified using GenomePlex® Single Cell Whole Genome Amplification Kit (Sigma-Aldrich). WGA-ChIP DNA was digested with BpmI (New England Biolabs) and 30 ng DNA was used for library preparation using NEBNext® ChIP-Seq Library Prep Master Mix Set for Illumina® (New England Biolabs). Library sequencing was performed on a HiSeq 3000 (Illumina).

Clean reads were obtained after removing low-quality reads by fastp software [2]. Then, clean reads were aligned to the human genome (hg38) with Bowtie2 (version 2.3.2) using default settings [3]. MACS2 was used to call significantly enriched peaks with default settings (-q 0.01) [4]. Then MAnorm3 were used to normalize read counts in common peaks between resistant and sensitive samples [5]. Overlapping peaks were merged by bedtools (version 2.27.0) between two similar samples [6]. We identified 40,424 (HANK1 sample) and 71,456 (SNK6 sample) H3K27ac peaks in chidamide-resistant samples. There were 42,739 (KHYG1 sample) and 30,924 (MEC04 sample) H3K27ac peaks identified in chidamide-sensitive samples. We merged the overlapping peaks between two resistant samples as resistant peaks (22,516) and between two sensitive samples as sensitive peaks (14,574). From 28,727 union peaks of H3K27ac in resistant and sensitive peaks, relative fold changes of each H3K27ac peaks from resistant samples were calculated by comparing with averaged H3K27ac signals of sensitive samples. With this, GAIN regions (9267 peaks) were defined as the union of H3K27ac peaks more than 2-fold increase of H3K27ac signal in any of resistant samples compared to sensitive samples while LOSS regions (3558

peaks) were defined as more than 2-fold decrease of H3K27ac signal. To annotate ChIP-seq peaks, the script annotatePeaks.pl from HOMER suites was used to determine whether a peak was in the promoter (TSS, transcription start site), exon (Coding), 5’ UTR exon, 3’ UTR exon, intron, or intergenic, and we also used this script to determine the distance to the nearest TSS and assigned the peak to that gene [7].

Real-time RT–qPCR, RNA-seq and RNA-seq data analyses

Cells were lysed using TRIzol™ Reagent (Thermo Fisher Scientific) and RNA was extracted using the RNeasy Mini Kit (Qiagen). Aliquots of 500 ng RNA were reverse-transcribed using TransScript® All-in-One First-Strand cDNA Synthesis SuperMix for qPCR (One-Step gDNA Removal) (Transgen Biotech). RT–qPCR was performed following the instructions of KAPA SYBR® FAST Universal 2X qPCR Master Mix (KAPA Biosystems).

1 μg total RNA was used for library preparation. Ribosomal RNA (rRNA) and fragmented RNA were removed by using TruSeq® Stranded RNA HT kit 96 samples Ribo-Zero™ Gold (Illumina). Library preparation was conducted according to TruSeq® Stranded Total RNA Sample Preparation Guide-Low Sample (LS) Protocol (Illumina). Library sequencing for 6 NKTL cell lines was performed using Hiseq 2500 (Illumina) and library sequencing for HANK1 treated with DMSO, tofacitinib or ruxolitinib was performed using Novaseq 6000 (Illumina).

Raw data were subjected to fastp software for quality checking and filtering of low-quality reads. Then, clean data were mapped to the human reference genome (hg38) using STAR v2.7.0f [8]. Separately, sample-specific expression analysis was performed by abundance estimation using RSEM [9]. Differentially expressed genes were identified using the Bioconductor software edgeR (p < 0.05). We used customized R scripts to generate visualizations such as MA plots, correlation plots and clustered heatmap. To create heatmap-density plot, the customized R script was used by centering peak regions extended to ± 5000 bp with 100 bp bins. Gene set enrichment analysis was based on hypergeometric test that takes the size of the overlap between the hallmark gene set and the list of differentially expressed genes overlapping with H3K27ac GAIN and LOSS regions in resistant versus sensitive cells as the test statistic. To generate browser tracks for visualization in IGV, the bamCoverage software from deepTools was used [10].

In vivo studies

Tumor volume was measured by Vernier caliper and calculated with the following formula: tumor volume (V) = width^2 × length × 0.537. A total of 2.5 x 10^6 NK-S1 cells were resuspended in 50 μl of PBS and 50 μl of Matrigel and injected subcutaneously into the left flank of each animal. When tumors reached ~100 mm^3, the mice were divided into four groups for treatment. Randomization was performed by equally dividing tumor-bearing mice of similar tumor burden into four groups for drug treatment. The mice were fed daily via oral gavage for 12 days with either

vehicle, 25 mg/kg of chidamide, 180 mg/kg of ruxolitinib or a combination of both chidamide and ruxolitinib. Tumor volume was monitored 3 times per week and body weight was measured daily until tumor volume reached 2000 mm^3. Mice were sacrificed by CO_2 inhalation and tumors were harvested for further analysis.

Supplementary References

1. Loong SL, Hwang JS, Lim ST, Yap SP, Tao M, Chong TW, et al. An Epstein-Barr virus positive natural killer lymphoma xenograft derived for drug testing. Leuk Lymphoma. 2008;49(6):1161-7.
2. Chen S, Zhou Y, Chen Y, Gu J. fastp: an ultra-fast all-in-one FASTQ preprocessor. Bioinformatics. 2018;34(17):i884-i90.
3. Langmead B, Salzberg SL. Fast gapped-read alignment with Bowtie 2. Nat Methods. 2012;9(4):357-9.
4. Zhang Y, Liu T, Meyer CA, Eeckhoute J, Johnson DS, Bernstein BE, et al. Model-based analysis of ChIP-Seq (MACS). Genome Biol. 2008;9(9):R137.
5. Shao Z, Zhang Y, Yuan GC, Orkin SH, Waxman DJ. MAnorm: a robust model for quantitative comparison of ChIP-Seq data sets. Genome Biol. 2012;13(3):R16.
6. Quinlan AR, Hall IM. BEDTools: a flexible suite of utilities for comparing genomic features. Bioinformatics. 2010;26(6):841-2.

7. Heinz S, Benner C, Spann N, Bertolino E, Lin YC, Laslo P, et al. Simple combinations of lineage-determining transcription factors prime cis-regulatory elements required for macrophage and B cell identities. Mol Cell. 2010;38(4):576-89.

8. Dobin A, Davis CA, Schlesinger F, Drenkow J, Zaleski C, Jha S, et al. STAR: ultrafast universal RNA-seq aligner. Bioinformatics. 2013;29(1):15-21.

9. Li B, Dewey CN. RSEM: accurate transcript quantification from RNA-Seq data with or without a reference genome. BMC Bioinformatics. 2011;12:323.

10. Ramirez F, Dundar F, Diehl S, Gruning BA, Manke T. deepTools: a flexible platform for exploring deep-sequencing data. Nucleic Acids Res. 2014;42(Web Server issue):W187-91.

Supplementary Figures

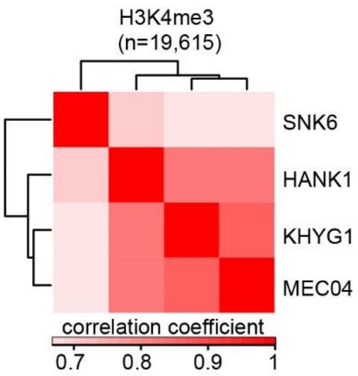

Fig. S1 H3K4me3 landscapes in chidamide-resistant and chidamide-sensitive NKTL cells. Heatmap representation of unsupervised hierarchical clustering based on H3K4me3 occupancy at total H3K4me3 ChIP-seq peaks (n = 19,615). Samples were clustered based on the Spearman correlation coefficient with average linkage.

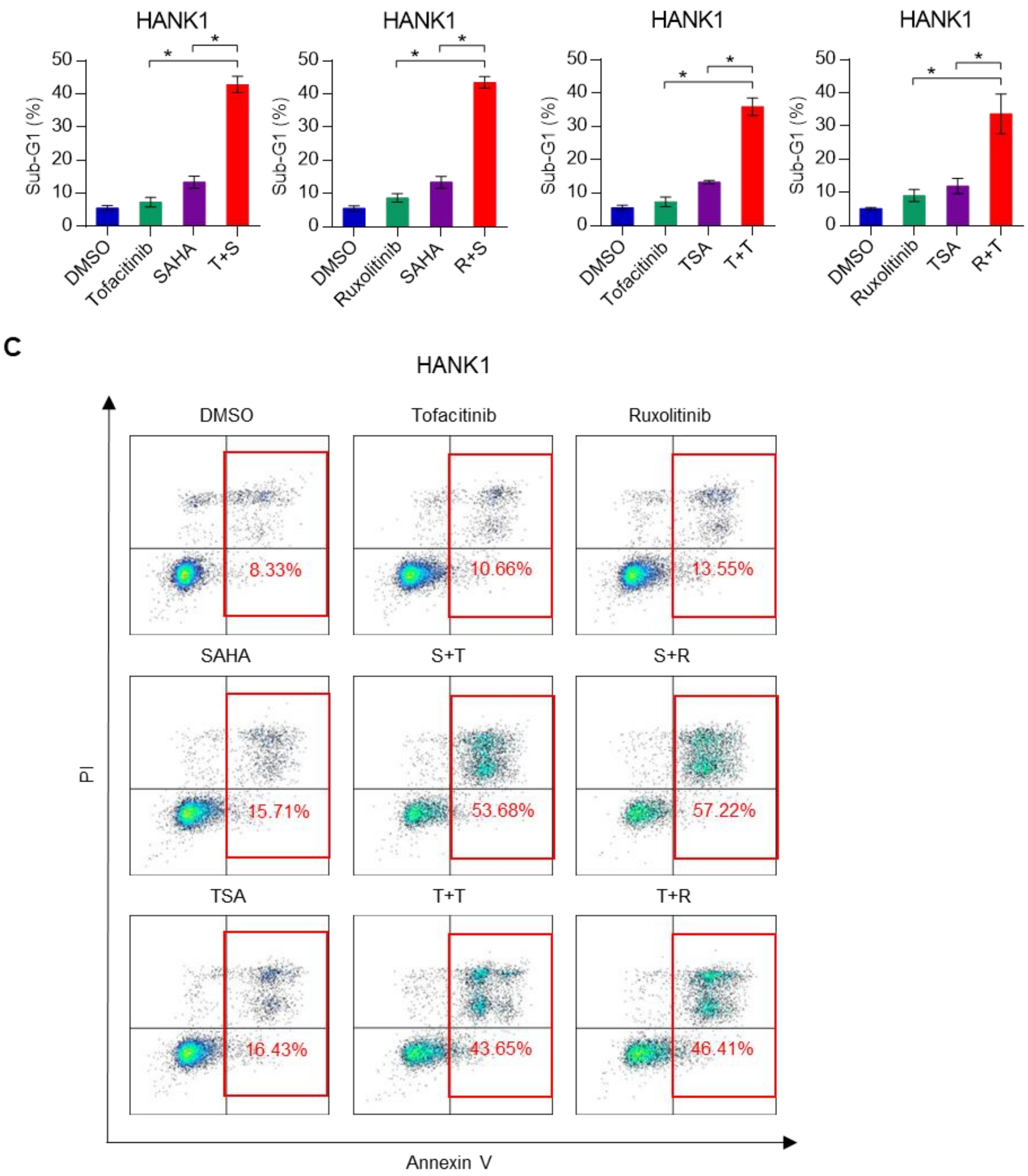

Fig. S2 JAK inhibitors overcome the resistance of SAHA/TSA in NKTL cells. **A** Cell cycle assay for the combination treatment of HANK1 cells with SAHA and tofacitinib/ruxolitinib for 72 hours. **B** Cell cycle assay for the combination treatment of HANK1 cells with TSA and tofacitinib/ruxolitinib for 72 hours. **C** Annexin V/PI staining of HANK1 cells treated with SAHA/TSA and tofacitinib/ruxolitinib for 72 hours. The results are expressed as the mean ± SD of three independent experiments.

* $p < 0.05$.
